# *Streptococcus sobrinus* as a Predominant Oral Bacteria Related to the Occurrence of Dental Caries in Polish Children at 12 Years Old

**DOI:** 10.3390/ijerph192215005

**Published:** 2022-11-15

**Authors:** Izabela Korona-Glowniak, Agnieszka Skawinska-Bednarczyk, Rafal Wrobel, Justyna Pietrak, Izabela Tkacz-Ciebiera, Monika Maslanko-Switala, Dorota Krawczyk, Adrian Bakiera, Anna Borek, Anna Malm, Maria Mielnik-Blaszczak

**Affiliations:** 1 Department of Pharmaceutical Microbiology, Medical University of Lublin, 20-093 Lublin, Poland; 2 Department of Paediatric Dentistry, Medical University of Lublin, 20-093 Lublin, Poland

**Keywords:** oral microbiota, *Streptococcus sobrinus*, dental caries, 12-year-old children

## Abstract

Dental caries is listed by the WHO as one of the major non-communicable diseases that need to be prevented and treated. The aim of the study was to evaluate the prevalence and severity of caries expressed as the Decayed, Missing and Filled Permanent Teeth (DMFT) index in 12-year-old Polish children and to verify bacterial species related to the occurrence of dental caries. Quantitative real-time PCR analysis of DNA isolated from saliva samples was performed to detect 8 cariogenic and periopathogenic bacterial strains. A total of 118 Polish children were enrolled in the study. They had low mean DMFT scores of 1.58 ± 1.98. The prevalence of dental caries in the children tested was low (53.4%), with a tendency to decrease compared to previous oral surveys. Bacterial abundance of other species in the dental caries and caries-free groups did not differ; however, periopathogenic *Prevotella pallens*, *Fusobacterium nucleatum* along with cariogenic *Streptococcus mutans* and *Lactobacillus fermentum* were significantly strongly correlated in the caries-active subjects. The prevalence of *S. sobrinus* was significantly higher in children with dental caries (*p* = 0.023) and correlated with higher DMFT. It may temporarily play an important role in the initiation of the cariogenic process or in its enhancement due to an ecological imbalance in dental microbiota.

## 1. Introduction

Dental caries is a considerable public health problem despite age. According to the World Health Organization (WHO), dental caries is a pathological process leading to decalcification of enamel, disintegration of hard tooth tissues and subsequent cavity formation [1]. Cavities are caused by acids produced from sugars by the oral bacteria present in the oral cavity. It is assumed that the development of the disease is determined by the occurrence of cariogenic bacteria fermenting carbohydrates into organic acids, mainly lactic acid [2]. The main factors of caries in children include cariogenic microorganisms, dietary supply of carbohydrates and susceptibility of the tooth surface to these factors [3,4].

For many years, little attention has been paid to dental caries. Currently, it is one of the main health problems in all age groups in developed countries [1,5,6]. It is estimated that cavities occur in 60–90% of children and in the vast majority of adults. In the US, dental caries is the most common chronic disease among children and is up to five times more common than asthma [5,6]. Therefore, dental caries is listed by the WHO, together with tumors and hypertension, as the three major non-communicable diseases that need to be mainly prevented and treated [7].

Although caries and periodontitis are clearly bacterial diseases, they are not infectious diseases in the classical sense because they result from a complex interaction between the commensal microbiota, host susceptibility and environmental factors such as diet and smoking. Therefore, oral health has been constantly monitored. Age reference groups of children aged 6, 12, 18 years and adults aged 35–44 and 55–64 years have been specified and subjected to regular dental check-ups. According to the principles of oral health monitoring adopted by the WHO, adolescents at 12 years old are examined every three years. The designated time intervals depend on the incidence of caries disease and its dynamics related to a particular stage of human life [7].

The oral cavity is colonized by a diverse range of bacteria. Due to their enormous amount, it is one of the largest bacterial communities in the body. Colonization begins in the perinatal period and affects all tissues in the oral cavity [5,8,9]. Tooth colonization by bacteria is associated with the formation of a biofilm. The relationship between the oral microbiota and the host is very dynamic. The presence of risk factors, as well as disorders in salivary secretion, may lead to disturbances in this connection and, as a consequence, the appearance of pathological biofilms and the development of caries [5,6,8]. The first and key stage for caries development is infection of the oral cavity with mutans streptococci. The next stage is the accumulation of a large number of microorganisms that metabolize at a low pH, which is associated with the dominance of cariogenic microorganisms in the oral cavity and on the surface of dental plaque. The final point for the formation of carious defects is the predominance of demineralization processes over remineralization in the enamel/plaque/clay interphase [4,10].

Three major hypotheses concerning the etiology of dental caries are considered. First, the specific plaque hypothesis suggests the key role of specific species, such as *S. mutans* and *S. sobrinus*, in caries development. The second is the non-specific plaque hypothesis, implying caries as the outcome of the overall activity of the total plaque microbiota. Finally, the ecological plaque hypothesis proposes that caries is a result of a shift in the balance in the resident microbiota activated by environmental changes [11]. Therefore, dental caries has no specific microbial etiology, as any species that has relevant properties can contribute to the development of the disease.

Early caries diagnostics, risk assessment and individualized caries prevention plans will allow control of the disease and achieve an intentional effect. For the dentist, the most important thing is not only to treat the consequences of the disease but also to be aware of dental caries as a biological phenomenon. The aim of the study was to evaluate the prevalence and severity of caries expressed as the Decayed, Missing and Filled Permanent Teeth (DMFT) index in 12-year-old Polish children and to verify which bacterial species were related to the occurrence of dental caries.

## 2. Materials and Methods

### 2.1. Study Population

The subjects were patients who were consulted by dentists from the Department of Paediatric Dentistry, Medical University of Lublin. One hundred eighteen 12-year-old school children with permanent dentition participated in the study. The protocol was reviewed and approved by the Bioethics Committee of the Medical University of Lublin (KE-0254/174/2017) and performed in agreement with the Helsinki declaration. Written informed consent was obtained from each patient. Additionally, this clinical study was conducted under the National Oral Health Surveillance in October and November 2019 and approved by the Polish Ministry of Health. Considering the size of the children population aged 12 years in the tested region is approximately 22,800, the margin of error of the measured value at the 95% confidence interval is ±8.98% when tested 118 children.

A dental examination was performed with a dental mirror and an overhead lamp. The children were checked for dental caries by previously trained and calibrated dentists. The number of teeth with dental caries, fillings and teeth lost as a result of the carious process were recorded. The caries scores of each participant were noted and the DMFT (Decayed, Missing and Filled Permanent Teeth) index was calculated. Teeth were classified for individual components of the index in accordance with WHO guidelines [12]. 

The following diagnostic criteria for dental caries were applied [13]:

- A decayed tooth (DT) defines a tooth in which a cavity could clearly be seen or a lesion could be felt with an explorer in a pit or fissure or on a smooth surface. It also includes temporary fillings in the teeth.
- Missing tooth (MT) defines a tooth that has been lost or extracted for dental caries, not at replacement age. Third molars were excluded.
- Filled tooth (FT) defines a tooth with one or more permanent restorations and no cavity anywhere on the tooth.

### 2.2. Sample Collection

The patients were instructed to refrain from the intake of any food or drink and from the use of a toothbrush or mouth rinse 1 h before sampling. For saliva collection, the Sallivette^®^ (Sarstedt, Nümbrecht, Germany) with plain cotton swabs was used. The patient removed the swab from the Salivette^®^ and placed the swab in the mouth and chewed it for about 60 s to stimulate salivation. Centrifugation for 10 min at 4500 rpm yielded a clear saliva sample in the conical tube. The average saliva volume recovered was 1.1 ± 0.3 mL. Saliva samples were stored at −70 °C until real-time PCR testing.

### 2.3. Genomic DNA Isolation: Positive Control Microorganisms

*Streptococcus mutans* ATCC 25175, ATCC 31989, *S. sobrinus* ATCC 33478, *Lactobacillus acidophilus* ATCC 4356, *Lactobacillus fermentum* ATCC 23271, *Aggregatibacter actinomycetemcomitans* ATCC 33380, *Fusobacterium nucleatum* ATCC 25586, *Veillonella parvula* ATCC 10790 and *Prevotella pallens* ATCC 700821 were used as the standards in this study. Genomic DNA from these strains was isolated and purified from an overnight culture of Brain Heart Infusion according to the manufacturer’s (Qiamp DNA Mini Kit). It was confirmed by 0.7% agarose gel electrophoresis that the extracted DNA had a high molecular weight and a single band. The nucleic acid concentration was determined spectrophotometrically. The calibration curve was generated separately for each gene of the reference bacterial species in 10-fold serial dilutions of the DNA template in separate real-time reactions to determine their threshold cycle values. 

### 2.4. Real-Time PCR Analysis

Genomic DNA purification with a QIAamp DNA Mini Kit (Qiagen, Germantown, MD, USA) was used for DNA extraction from saliva samples according to the manufacturer’s instructions. Identification of bacteria was performed with 8 pairs of specific primers (Table S1) [14,15,16,17,18] in Real-Time PCR (LightCycler 96, Roche, Basel, Switzerland). The quantity of each experimental sample was first determined using a standard curve and then expressed relative to the calibrator sample.

### 2.5. Statistical Analysis

The presence/absence of bacteria was analyzed in relation to clinical conditions using the χ^2^ or the Fisher exact test, and the prevalence ratio and confidence interval were calculated. Data from qPCR quantification of bacterial species levels per milliliter of saliva were transformed into log numbers for statistical analysis. The Mann–Whitney test was used to compare bacterial counts from qPCR with the clinical parameters. The power and direction of the relationships between pairs of continuous variables were determined using Spearman’s coefficient of rank correlation. The significance level was set at 5% (*p* < 0.05).

## 3. Results

A total of 118 Polish 12-year-old children (61 males and 57 females) were enrolled in the study; no subjects had missing teeth caused by dental caries. They had mean DT and DMFT scores of 1.47 ± 1.9 and 1.58 ± 1.98, respectively. There were no significant differences in DMFT scores between males (1.39 ± 1.86) and females (1.79.3 ± 2.1; *p* = 0.36) (Table 1). Among the subjects, 63 children were diagnosed with dental caries (31 males and 32 females). The prevalence of dental caries was 53.4%. However, a higher number of girls with dental caries detected no significant difference between the sexes (*p* = 0.58, OR 1.24, 95%CI 0.6–2.6).

**Figure 1:**
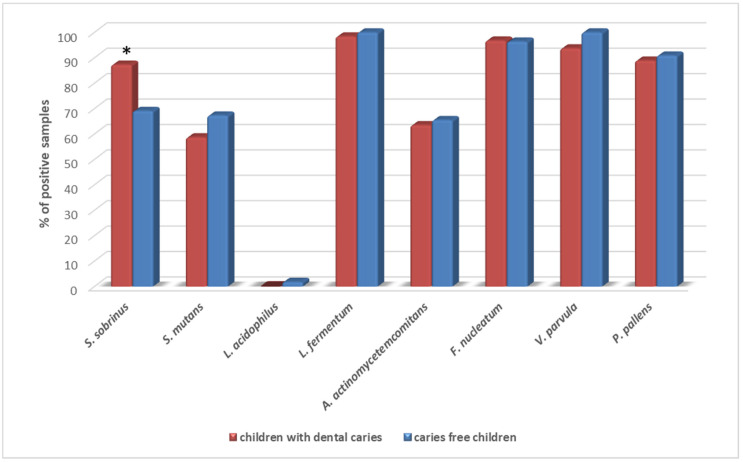
Prevalence of the tested bacterial species in saliva samples of children with dental caries and caries-free children. * *p* = 0.023 (Fisher’s exact test: children with dental caries vs. caries-free children).

The most prevalent pathogens detected in patients were *L fermentum* (99.2%), *F. nucleatum* (96.6%), *V. parvula* (96.6%) and *P. pallens* (53.3%). *L. acidophilus* was detected in only one patient. *S. sobrinus, S. mutans* and *A. actinomycetemcomitans* were found in 78.8%, 64.4% and 62.7%, respectively. Only the prevalence of *S. sobrinus* was significantly higher in children with dental caries diagnosed (*p* = 0.023, RR 1.8, 95% CI 1.0–3.35) (Figure 1).

**Figure 2:**
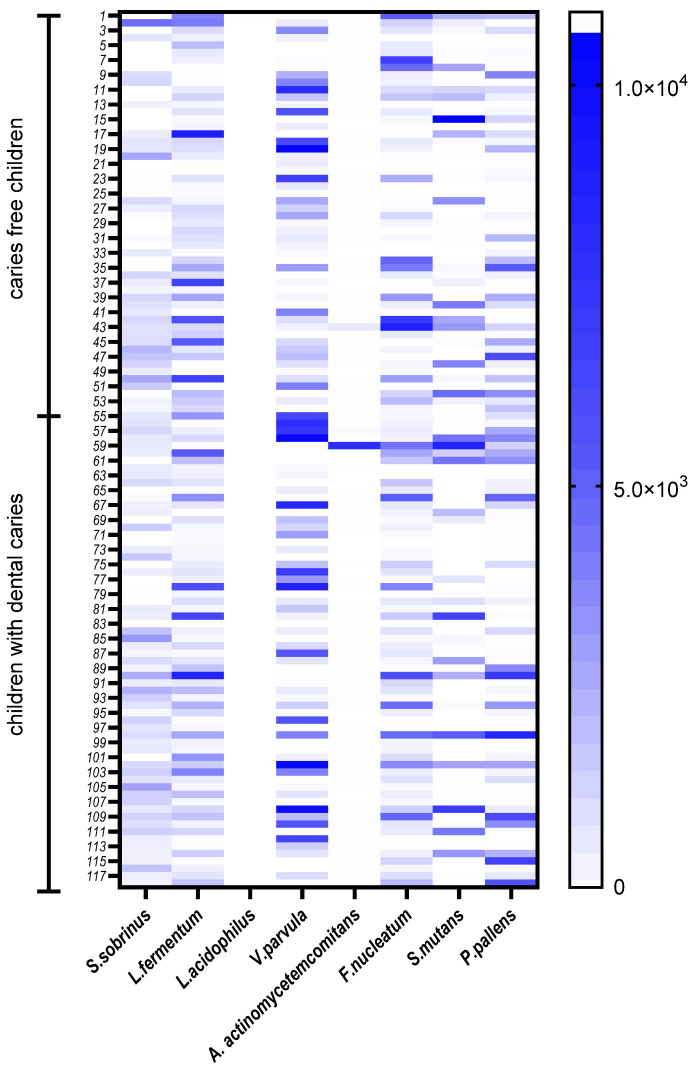
The relative abundance of species genes in the tested children from saliva samples presented on the heat map.

The abundance of selected oral bacterial species is presented in Figure 2. *Veillonella parvula* was the most abundant species, followed by *Lactobacillus fermentum, S. sobrinus* and *F. nucleatum* (Table 2). The comparison of the median values of each pathogen according to gender is shown in Table 3. A higher abundance of *S. sobrinus* and *S. mutans* was found in saliva samples from girls, whereas *V. parvula, P. pallens* and *L. fermentum* were found in higher quantities in samples from boys. A significantly lower amount of *S. sobrinus* was observed in the samples from boys (*p* = 0.004). No significant differences were observed by gender for the other species. Moreover, *S. sobrinus* was positively correlated with a higher DMTF index in the children tested (Table 3).

**Figure 3:**
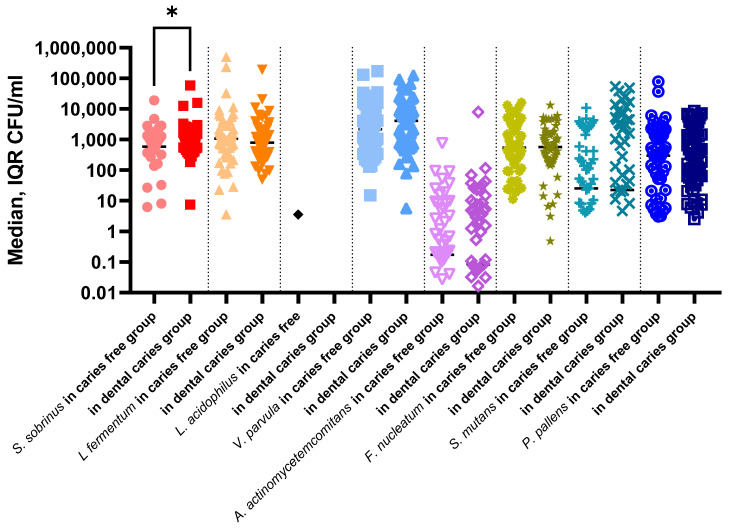
Abundance of bacterial species in saliva samples in the dental caries group and caries free group. (* *p* = 0.032, Mann–Whitney U Test: children with dental caries vs. caries free children).

The comparison of the median values of each pathogen according to dental caries occurrence is shown in Figure 3. A significantly higher abundance of *S. sobrinus* was observed in children with dental caries than in healthy children. It was also confirmed in a statistically significant positive correlation of higher *S. sobrinus* abundance with a higher DMFT index (Table 3).

**Figure 4:**
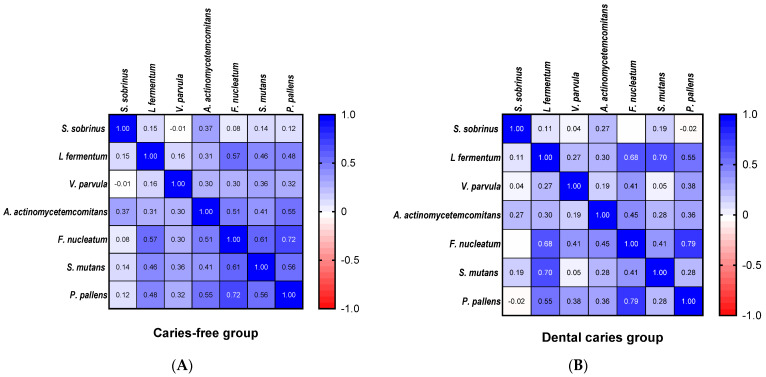
Correlation plot of the abundance of bacterial species. (**A**) caries free group, (**B**) dental caries group. (Spearman rank-order correlations test).

In the correlation analysis, *S. sobrinus* was positively associated with *A. actinomycetemcomitans* regardless of decay status (Figure 4). In fact, the occurrence of most tested bacterial oral species was significantly correlated. However, a strong correlation in the caries-free group was found for more species in comparison to the dental caries group. In the first group, *F. nucleatum* had a positive relationship with *P. pallens*, *S. mutans*, *L. fermentum* and *A. actinomycetemcomitans*. In the dental caries group, the strongest relationships were observed for *L. fermentum, S. mutans, F. nucleatum* and *P. pallens*.

## 4. Discussion

Children under the age of 12 are among one of the groups of great interest for the WHO epidemiological measurement of caries intensity and, thus, one of the most studied age groups for the evaluation of oral health [7,13]. At this age, all permanent teeth except the third molars will have erupted and they are easily available for dental examination by the primary school system. Therefore, the age of 12 years was selected as the indicator age for global comparison. Several articles have described the dental caries status of 12-year-old children based on data from a single national survey [19,20,21,22,23,24]. A DMFT index of less than 1.2 is judged to be very low, 1.2–2.6 is low, 2.7–4.4 is moderate, and 4.5 or more is high. To effectively prevent the development of dental caries, it is important to determine appropriate preventive measures and identify risk factors [3].

A vast majority of countries were able to meet the WHO target of no more than three DMFT by the year 2000 (WHO, 2003). Poland is one of the few countries in Europe that failed to meet the assumptions of the second oral health objective for 2000 [25]. The studies from Poland in 2005 showed a high value of the caries intensity index to the DMFT value = 3.3, which seems to be related to the liquidation of dentist offices in schools and a reduction in the availability of dental services. The dental caries parameters in Poland in 1995–2012 indicated some uneven decreasing trends, with the highest caries frequency (90.5%) and DMFT value (4.3) in 1995 and the lowest caries frequency (79.6%) in 2012 and DMFT in 2007 (3.07) [23]. In the next years, the DMFT index was continuously decreasing to 2.8 in 2014 (https://capp.mau.se/country-areas/poland/ (accessed on 6 September 2022)). Rybarczyk-Townsend et al. reported a decrease in dental caries by 1.4% in 12-year-old children in Łódzkie region (67.8%), and the DMFT index decreased to 1.63 [26]. Similarly, the DMFT index in this study was 1.58. Surprisingly, among 12-year-olds in Skierniewice, Poland, in 2020, the DMFT index was twice as high—3.8 [22]. The WHO objective for 2020, to reduce the proportion of adolescents aged 13 to 15 years with dental caries experience in their permanent teeth to 48.3%, failed in Poland, unfortunately. In this study, the prevalence of dental caries was 53.4%, almost equal to the value of baseline—53.7% of adolescents aged 13 to 15 years who had dental caries experience in at least one permanent tooth in 1999–2004 [12]. 

Dental caries etiology is a multifactorial interaction of four primary factors: host factors, cariogenic microorganisms growing on favorable substrates, and a low pH exposition time [2,27]. Children are particularly susceptible to this disease due to the low degree of enamel and dentin mineralization and thus are more vulnerable to infection caused by cariogenic bacteria. One of the main goals facing researchers is to correlate specific microbial species or communities with dental caries. The very first step in identifying the potential microbial factors is precisely describing the composition of the oral microbiota, which is critical in dental health and disease [28]. Recently, bacterial identification, listing and the particular role of microorganisms in health and disease have received increasing attention. 

In this study, real-time PCR analysis of the saliva microbiota of caries-free healthy adolescents and dental-caries adolescents was carried out. All pathogens considered in our study were previously identified in oral samples of children or adolescents [5,10,28,29]. Along with cariogenic bacteria, some periodontopathogenic species were also identified to make the study more complex. In fact, some studies have demonstrated a positive association between periodontitis and caries, but others are contradictory [30,31].

*Streptococcus mutans* is generally recognized as a bacterial species responsible mainly for caries decay [32]. However, it has been noted that caries can occur even in the absence of an *S. mutans* count. Moreover, it has been found that the amount of *S. mutans* is low even when caries is found, suggesting that other species may also be involved in caries development [33,34]. In this study, similar *S. mutans* loads were found in both caries-free and dental caries active groups, without a significant difference. Tanner et al. [29] reported that *S. mutans* was not detected in any of the subjects in the caries-active group. Moreover, *S. mutans* was also detected in supragingival plaque from caries-free children. These observations are consistent with data from this study, in which 67% of caries-free children were colonized by *S. Mutans*, while in 41% of caries-active groups, *S. mutans* was not detected. According to another report, *S. mutans* was not identified in all types of carious lesions or all white spots, despite the use of culture or molecular methods [29]. 

The second cariogenic species, *S. sobrinus,* was detected in this study significantly more frequently and in higher amounts in the dental caries group. Moreover, a positive correlation between *S. sobrinus* loads and higher DMFT values was observed. The correlation of the presence of *S. sobrinus* with high caries experience was also observed in other studies [35]. Although several studies have shown that children who carried both *S. mutans* and *S. sobrinus* had higher caries experience, caries incidence and total counts of mutans streptococci than children carrying only *S. mutans* [36,37], this was not observed in this study. In our study, in all children, *S. mutans* and *S. sobrinus* were detected in 61.9% and 78.8%, respectively, whereas 13.6% were positive for *S. mutans* alone, 30.5% for *S. sobrinus* alone, and 48.3% for both *S. mutans* and *S. sobrinus*, with 7.6% being negative for both streptococci. Similarly, in other studies, *S. sobrinus* was also the predominant species [38]. These results indicate that the prevalence of mutans streptococci in 12-year-old Polish children is 92.4%, which is very high and consistent with similar surveys conducted in other parts of the world [14,38,39]. Nevertheless, some studies indicate that the relation between *S. mutans* and caries is not fundamental because *S. mutans* count can colonize tooth surfaces without injury occurring and caries can develop in the absence of this species, which was also shown in our study [40]. This study revealed a significant association between *S. sobrinus* and dental caries. The findings of Rupf et al. [41] suggest that an *S. sobrinus* infection represents an important additional risk factor for dental caries due to its obvious aggravating of caries activity. Moreover, Hughes et al. reported that baseline counts of *S. sobrinus*, but not *S. mutans*, were higher in children with recurrent compared with no recurrent caries [42]. Gross et al. [43] showed that *S. sobrinus*, unlike *S. mutans*, was not detected in any healthy control subjects and was probably a more effective factor in caries than *S. mutans*. Fragkou et al. [37] reported that nearly all 3-to-13-year-old children were found to carry *S. mutans* and *S. sobrinus,* with the proportions of *S. sobrinus* to be higher than those of *S. mutans*, both in dental plaque and saliva and their presence was statistically significantly related to the caries experience. Kneist et al. [44] assessed the virulence of acidogenic and aciduric oral streptococci in an in vitro caries model using penetration depths into dental enamel. After 10 weeks of incubation, the invasion of *S. sobrinus* reached depths of 87.53 ± 76.34 μm below the break edges into the enamel, while the penetration of *S. sanguinis* was 11.13 ± 24.04 μm. 

*Lactobacillus* spp. are highly acid-tolerant anaerobes, thriving in the acidic environment on active dentin carious lesions. *Lactobacillus* spp. were reported to significantly increase in the caries-active group [45]. Although in the current study, *L. fermentum* was one of the most frequent and abundant species, no significant difference was found between caries-free and dental-caries active groups. Formerly, *L. salivarius* was found practically exclusively in dentin caries, suggesting that this species might be more significant in the advanced stages of caries [28,29,46]. Interestingly, in another study, *Lactobacillus* spp. was detected in caries-free subjects [45], similar to the present study, where *L. fermentum* was found in comparable loads in caries-free children as well as in children with dental caries. In previous studies, *Lactobacillus fermentum* was the most common species isolated from carious lesions in Thai children [47]. The results for *L. acidophilus,* which were almost absent in the present study, are similar to previous reports and could not be correlated with caries [10,48].

Taking into account that over 19,000 phylotypes may inhabit the oral cavity, molecular techniques have bridged the gap in the identification of uncultivable or difficult-to-culture microorganisms, allowing most in-depth, comprehensive and collaborated views to date of the oral microbiota in both caries-active and carries-free individuals [34]. Among the other bacteria, non-mutans streptococci and bacteria from genera such as *Actimomyces, Bifidobacteria, Neisseria* and *Veillonella* are enumerated [33]. 

The involvement of *Veillonella* spp. in caries development is not well understood. The abundance of *V. parvula* in this study showed no differences in its prevalence in relation to caries status. Gross et al. [49] reported no significant relationship between caries status and *Veillonella* spp. presence, whereas other investigators showed a significant association between *Veillonella* spp. increase and caries progression [28]. 

In this study, we detected qualitatively and quantitatively bacteria commonly classified as cariogenic—*Lactobacillus* spp., *S. mutans, S. sobrinus*; bacteria considered peripathogenic—bacteria from the purple complex (*V. parvula*), the green complex (*A. actinomycetemcomitans*), and the orange complex (*F. nucleatum, P. pallens*). In the dental caries group, the strongest co-occurrence of orange complex bacteria and cariogenic *S. mutans* and *L. fermentum* was found. Although the same species were positively associated in samples from caries-free children, the correlation was less strong and extended to more tested species. It seems that this study is consistent with a recent study on childhood caries that reported no difference between caries-active and caries-free saliva microbiotas using PCR-based denaturing gradient gel electrophoresis and pyrosequencing [50]. Dental caries is the result of an ecological imbalance of the dental plaque and some authors consider that species-level resolution for caries prognosis is underscored [10]. In our findings, an increase in *S. sobrinus* detected in the dental caries group may play an important role in the initiation of the cariogenic process or in its enhancement, as was shown elsewhere [41]. However, we believe that our findings were rather consistent with the ecological hypothesis and this study could reveal only temporary conditions that prefer an increase of *S. sobrinus* as an example of the microbial imbalance. It also confirms the importance of this cariogenic species in decay development. Our research should be extended to correlate more microbial species with cariogenesis progression and should be tested on larger populations of children.

The main limitation of this study was that we collected only saliva samples from the patients. The bacterial composition could have been different if samples from plaque were included. Even though there are studies on saliva-based carries risk assessment models [10,16,17], it was confirmed that the composition and diversity of the oral microbiome differed significantly from the types of samples collected, i.e., saliva and plaque [51,52]. For example, it was noted that *Streptococcus mutans* was detected in children aged 6–12 years with dental caries in both dental plaque and saliva samples, while *Streptococcus sobrinus* was enriched in this group in saliva samples only [52].

## 5. Conclusions

However, the prevalence of dental caries in 12-year-old children in Poland was not low, with a tendency to decrease compared to previous oral surveys. Although there were no differences in bacterial abundance in the dental caries and caries-free groups, this study found that the periodontopathogenic species *P. pallens, F. nucleatum* along with cariogenic *S. mutans* and *L. fermentum* were significantly correlated in the dental caries subjects. In our findings, an increase in *S. sobrinus* detected in the dental caries group may play an important role in the initiation of the cariogenic process or in its enhancement. Nevertheless, this confirms the ecological hypothesis of dental caries development and the temporary imbalance in dental microbiota. 

## Figures and Tables

**Table 1:** Caries intensity expressed by the DMFT index with DT, MT and FT components in the 12-year-olds according to gender (mean values ± standard deviations).

Variables	Total (*n* = 118)	Boys (*n* = 61)	Girls (*n* = 57)	*p* Value ^1^
DT	1.47 ± 1.9	1.21 ± 1.7	1.74 ± 2.07	0.24
MT	0	0	0	-
FT	0.12 ± 0.47	0.18 ± 0.59	0.05 ± 0.29	0.17
DMFT	1.58 ± 1.98	1.39 ± 1.86	1.79 ± 2.07	0.36

DT, Decayed tooth; MT, Missing tooth; F, Filled tooth; DMFT, the mean number of caries (D  +  M  +  F). ^1^ Mann–Whitney U Test: boys vs. girls.

**Table 2:** Relative bacterial abundance of saliva samples in relation to gender in saliva samples.

Bacterial Species	Total (*n* = 118)	Girls (*n* = 57)	Boys (*n* = 61)	*p* Value
	Median (IQR)			
*S. sobrinus*	661.85 (32.85–1272.0)	937.6 (553.1–1482.0)	539.8 (0.0–918.2)	0.0039 *
*L. fermentum*	934.0 (316.0–2103.0)	738.2 (270.3–2816.0)	986.2 (393.3–1867.0)	0.88
*V. parvula*	2823.0 (615.6–12080.0)	2174.0 (488.1–12080.0)	4054.0 (730.7–10970.0)	0.35
*A. actinomycetemcomitans*	0.13 (0.0–4.93)	0.54 (0.0–8.11)	0.077 (0.0–2.67)	0.17
*F. nucleatum*	569.6 (164.4–1561.0)	537.0 (144.5–1496.0)	637.0 (200.6–1693.0)	0.58
*S. mutans*	24.25 (0.0–2197.0)	43.6 (0.0–3121.0)	19.9 (0.0–632.9)	0.22
*P. pallens*	281.2 (21.21–1448.0)	144.8 (7.8–1167.0)	401.9 (64.6–2156.0)	0.13

* statistically significant (Mann–Whitney U Test: boys vs. girls), IQR, interquartile range.

**Table 3:** Correlation of bacterial species abundance with the DMFT index.

DMFT Index in Correlation to:	Spearman R	t (N − 2)	*p*-Value
*S. sobrinus*	0.26	2.91	0.0043
*L. fermentum*	−0.044	−0.48	0.63
*L. acidophilus*	−0.093	−1.01	0.32
*V. parvula*	0.093	0.98	0.33
*A. actinomycetemcomitans*	0.095	1.02	0.31
*F. nucleatum*	0.072	0.78	0.44
*S. mutans*	0.1	1.09	0.28
*P. pallens*	0.032	0.35	0.73

DMFT (Decayed, Missing, and Filled Permanent Teeth).

## Data Availability

Due to privacy and ethical concerns, the data that support the findings of this study are available on request from the Last Author [M.M.-B.].

## Supplementary Materials

The following supporting information can be downloaded at: https://www.mdpi.com/article/10.3390/ijerph192215005/s1.
