# Supplementary material for: Streptococcus sobrinus as a Predominant Oral Bacteria Related to the Occurrence of Dental Caries in Polish Children at 12 Years Old
Source: Int J Environ Res Public Health. 2022 Nov 15;19(22):15005. doi: 10.3390/ijerph192215005 (PMC9690266; doi:10.3390/ijerph192215005)
Supplement: Supplementary file 1 [file ijerph-19-15005-s001.zip › ijerph-1984786-supplementary.pdf]

Table S1. Sequence of primers used in real-time PCR.

| Species | Sequence (5'-3') | Product size (16S rRNA) | reference |
|---|---|---|---|
| *Streptococcus mutans* | | | |
| Forward primer | GCAGTCAAGGGGTGGAAATCG | 188 bp | 14 |
| Reverse primer | TGGACGGCTTGTTGCAGGAATAC | | |
| *Streptococcus sobrinus* | | | |
| Forward primer | AAAACATTGGGTTACGATTGCG | 156 bp | 14 |
| Reverse primer | CGTCATTGGTAGTAGCCTGA | | |
| *Lactobacillus acidophilus* | | | |
| Forward primer | GAAAGAGCCCAAACCAAGTGATT | 85 bp | 15 |
| Reverse primer | CTTCCCAGATAATTCAACTATCGCTTA | | |
| *Lactobacillus fermentum* | | | |
| Forward primer | TGGAAACAGRTGCTAATACCG | 231 bp | 16 |
| Reverse primer | GTCCATTGTGGAAGATTCCC | | |
| *Aggregatibacter actinomycetemcomitans* | | | 17 |
| Forward primer | GAACCTTACCTACTCTTGACATCCGAA | 80 bp | |
| Reverse primer | TGCAGCACCTGTCTCAAAGC | | |
| *Fusobacterium nucleatum* | | | |
| Forward primer | GGATTTATTGGGCGTAAAGC | 163 bp | 17 |
| Reverse primer | GGCATTCCTACAAATATCTACGAA | | |
| *Prevotella pallens* | | | |
| Forward primer | AACGAATGAATGTCCGCGATAATGAAGTC | 190 bp | 18 |
| Reverse primer | CGAGAAAGACCACCTGGACCTAAAGCA | | |
| *Veillonella parvula* | | | |
| Forward primer | GAACGTTTGTTGCGTGCTATTTTTGGT | 128 bp | 18 |
| Reverse primer | TCGTCGCCATTTTCACGGGTAA | | |
